# Chemical Transdifferentiation of Somatic Cells: Unleashing the Power of Small Molecules

**DOI:** 10.3390/biomedicines11112913

**Published:** 2023-10-27

**Authors:** Yu Zhang, Xuefeng Li, Jianyu Xing, Jinsong Zhou, Hai Li

**Affiliations:** 1 Department of Histology and Embryology, School of Basic Medical Sciences, Xi’an Jiaotong University, Xi’an 710061, China; yuzhang@cau.edu.cn; 2 Department of Pathogenic Microbiology and Immunology, School of Basic Medical Sciences, Xi’an Jiaotong University, Xi’an 710061, China; lixuefeng57@126.com; 3 The First Affiliated Hospital of Harbin Medical University, Harbin Medical University, Harbin 150006, China; xingjianyu1987@126.com

**Keywords:** transdifferentiation, epithelial–mesenchymal transition, cell reprogramming, small molecule

## Abstract

Chemical transdifferentiation is a technique that utilizes small molecules to directly convert one cell type into another without passing through an intermediate stem cell state. This technique offers several advantages over other methods of cell reprogramming, such as simplicity, standardization, versatility, no ethical and safety concern and patient-specific therapies. Chemical transdifferentiation has been successfully applied to various cell types across different tissues and organs, and its potential applications are rapidly expanding as scientists continue to explore new combinations of small molecules and refine the mechanisms driving cell fate conversion. These applications have opened up new possibilities for regenerative medicine, disease modeling, drug discovery and tissue engineering. However, there are still challenges and limitations that need to be overcome before chemical transdifferentiation can be translated into clinical practice. These include low efficiency and reproducibility, incomplete understanding of the molecular mechanisms, long-term stability and functionality of the transdifferentiated cells, cell-type specificity and scalability. In this review, we compared the commonly used methods for cell transdifferentiation in recent years and discussed the current progress and future perspective of the chemical transdifferentiation of somatic cells and its potential impact on biomedicine. We believe that with ongoing research and technological advancements, the future holds tremendous promise for harnessing the power of small molecules to shape the cellular landscape and revolutionize the field of biomedicine.

## 1. Introduction

Transdifferentiation, also known as direct lineage conversion, involves the conversion of one specialized cell type into another without passing through an intermediate stem cell state [1]. Unlike traditional approaches utilizing pluripotent stem cells to generate differentiated cell types, transdifferentiation enables the direct conversion of readily available somatic cells. This technique circumvents the ethical concerns and technical challenges associated with pluripotent stem cells while offering a more straightforward and efficient path to cell replacement therapies [2,3]. 

The concept of transdifferentiation emerged in the late 20th century when researchers discovered that cells within the same lineage could be converted into different cell types under specific conditions [4,5,6]. It was in 2006 that the field gained momentum with the landmark discovery of induced pluripotent stem cells (iPSCs) by Shinya Yamanaka and his colleagues [7]. iPSCs, derived from somatic cells through a reprogramming process, possess the ability to differentiate into any kind of cells. This breakthrough not only revolutionized the study of cell biology but also laid the foundation for exploring the transdifferentiation of somatic cells. Since then, researchers have made significant strides in transdifferentiation techniques, successfully converting fibroblasts into neurons, hepatocytes, cardiomyocytes, endothelial cells and even pancreatic beta cells, which have potential value for the treatment of neurodegenerative disorders, liver diseases, heart failure and diabetes [8,9,10,11].

In this review, we summarized the current progress and future perspective of the chemical transdifferentiation of somatic cells and further discussed its potential impact on biomedicine and the limitations hindering its clinical application. 

## 2. Methods for Cell Transdifferentiation 

Transdifferentiation can be achieved through various methods, including the earliest overexpression of lineage-specific transcription factors along with the modulation of signaling pathways [12]. By manipulating transcription factors, scientists can drive the conversion of one cell type to another, and by modifying signaling pathways involved in cell fate determination, they can further enhance the efficiency and specificity of transdifferentiation. Here we summarize and compare some commonly used techniques for cell transdifferentiation (Table 1).

### 2.1. Transcription Factor Overexpression

This method involves the introduction of specific transcription factors or regulatory molecules into target cells, thereby inducing the conversion of cell fate [3]. After the first report of the conversion of mouse embryonic fibroblasts into myoblasts by forced expression of MyoD, Yamanaka and his colleagues reprogramed somatic cells into iPSCs by introducing a combination of transcription factor Oct4, Sox2, Klf4 and c-Myc via viral vector [7,13,14]. In recent years, some novel genome editing tools, such as transcription activator-like effector nucleases (TALENs), Zinc Finger Nucleases (ZFNs) and CRISPR/Cas9 have been used to precisely modify the DNA sequence of target cells [15,16,17]. By introducing specific genetic modifications, such as gene knockouts, gene insertions or gene replacements, it is possible to reprogram cells into a desired cell type. Basically, this method has a high efficiency, but genetic manipulation and potential risk of tumorigenicity are still concerns for clinical applications.

### 2.2. Chemical Small Molecules

Chemical small molecules can be used to induce cell transdifferentiation by modulating various signaling pathways and epigenetic modifications [18]. These molecules can directly activate or inhibit specific signaling pathways to facilitate the acquisition of desired cell fate. Small molecules have been shown to successfully reprogram human fibroblasts to cardiac cells, neurons and even pluripotent stem cells by inducing changes in transcriptional programs [19,20,21,22]. Compared with viral vector-based reprogramming methods, small molecules have the advantages of being non-immunogenic, not viable for genetic manipulation and are easy to standardize, but identifying an optimal combination of small molecules requires a lot of effort [18]. 

### 2.3. MicroRNA-Based Reprogramming

**Figure 1:**
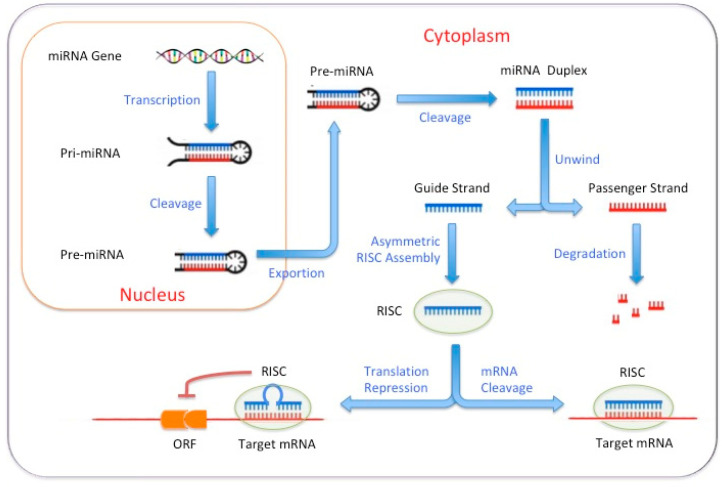
miRNAs regulate target mRNAs by disrupting their stability or inhibiting their translation.

MicroRNAs are small non-coding RNAs that play a role in the post-transcriptional regulation of gene expression. miRNAs bind and regulate target mRNAs by disrupting their stability or inhibiting their translation, depending on the matching degree with mRNA sequences. By introducing specific miRNAs into cells, it drives the transdifferentiation process and promotes the acquisition of a new cell fate by regulating their target genes (Figure 1). For example, the forced expression of miR-9 and miR-124 converted human fibroblast into neurons, and the combined overexpression of miR-1, miR-133, miR-208 and miR-499 has been used to reprogram cardiac non-myocytes into functional cardiac myocytes in vitro and in vivo [23,24].

### 2.4. Extracellular Vesicle (EV)-Based Reprogramming

EVs are small membrane-bound vesicles released by cells containing multiple bioactive molecules, including proteins, nucleic acids and lipids [25]. They serve as vehicles for intercellular communication by transferring functional molecules from one cell to another [26]. Recent studies have shown that EVs derived from specific cell types can induce transdifferentiation in the recipient cells [27,28]. Although the exact mechanisms underlying the transdifferentiation-inducing effects of EVs are still being investigated, it is believed that the transferred molecules can modulate gene expression and signaling pathways in the recipient cells, leading to changes in the cell phenotype and function. EV-based reprogramming, utilizing environmental cues and signaling factors, represents a safe and stepwise approach, but its limited efficiency and reproducibility are obstacles for further research and application.

Other than the methods mentioned above, there are also some rarely used ones, such as 3D cultivation and co-culture. We summarized the advantages and shortcomings of the methods for cell transdifferentiation in Table 1. Practically, a combination of different methods is employed to enhance the efficiency and effectiveness of cell transdifferentiation. For example, a combination of transcription factors, small molecules and miRNAs can be used to derive cardiac cells efficiently [29,30]. While it is worth noticing that although scientists continue to explore innovative approaches to reprogram cells and harness their regenerative potential, the specific approach depends on the cell type and the desired outcome.

## 3. Chemical Transdifferentiation of Somatic Cells 

### 3.1. The Power of Small Molecules: Chemical Transdifferentiation

One cutting-edge technique that has gained significant attention is chemical transdifferentiation, which enables researchers to bypass the traditional methods of cell reprogramming, such as genetic manipulation and viral delivery, by utilizing small molecules as powerful tools [8]. These molecules can directly modulate cellular signaling pathways and gene expression, leading to the desired cellular conversion. 

Initially, scientists focused on genetic techniques to initiate cell fate changes. Although this method yielded significant advancements, it also posed challenges such as potential genomic integration and difficulty in delivering large size genetic materials to the target cells [31,32]. Chemical transdifferentiation presents an alternative approach that overcomes these limitations. Small molecules, acting as signaling pathway modulators or epigenetic regulators, can directly influence the cellular state and promote the transition from one cell type to another [33,34]. By identifying the key molecular players involved in cell fate determination, scientists can design and optimize small molecule cocktails that trigger the desired cellular conversion. Until now, researchers have successfully employed this technique to convert various cell types across different tissues and organs, and in the later paragraphs, we will discuss this topic in detail. 

In fact, chemical transdifferentiation offers several significant advantages over other reprogramming methods, including simplicity, scalability and convenience for controlling the reprogramming process. 

(1) Simplicity and Accessibility: Genetic approaches, such as introducing transcription factors or viral delivery, often require intricate and complex procedures [31,32,33]. In contrast, small molecules are relatively easy to synthesize, manipulate and deliver to target cells. Their simplicity and accessibility make chemical transdifferentiation a more scalable and widely applicable method in both research laboratories and potential clinical settings.

(2) Precision and Temporal Control: Chemical transdifferentiation offers a unique advantage in terms of precision and temporal control over the reprogramming process [35]. Researchers can fine-tune the concentration, duration and timing of small molecule treatments to optimize efficiency and specificity. The convenience for controlling enhances the reproducibility and reliability of the technique, enabling scientists to generate the desired cell types with greater accuracy. Moreover, temporal control allows for stepwise or sequential transdifferentiation, mimicking natural development and facilitating the generation of complex cell populations [36].

(3) Versatility and Range of Applications: Chemical transdifferentiation demonstrates exceptional versatility, allowing for the conversion of a wide range of cell types across various tissues and organs. From fibroblasts to neurons, cardiomyocytes, hepatocytes, pancreatic beta cells et al., small molecules have successfully induced their transdifferentiation [19,37,38,39,40]. This broad applicability opens up a myriad of possibilities for regenerative therapies, disease modeling, drug discovery and personalized medicine. 

(4) Patient-Specific Approaches: Chemical transdifferentiation holds immense potential for personalized medicine [37]. By utilizing patient-derived cells, it becomes possible to generate specific cell types for each individual, minimizing the risk of immune rejection and enhancing therapeutic outcomes [38,39]. This personalized approach has the potential to revolutionize treatments by providing patient-specific therapies that are tailored to the unique needs of individuals. Additionally, it allows for the study of disease mechanisms using patient-specific cells, paving the way for more accurate disease modeling and drug screening.

(5) Ethical and Safety Considerations: Unlike techniques involving pluripotent stem cells, chemical transdifferentiation bypasses ethical concerns related to embryo usage or genetic modification. By utilizing small molecules and readily available somatic cells, this approach offers a more ethically and socially acceptable alternative for cell reprogramming. Moreover, since the cells are generated directly from the patient’s own tissues, the risk of immune rejection is significantly reduced, minimizing the need for immunosuppressive therapies [39].

(6) Translational Potential: Chemical transdifferentiation shows great promise for translational applications in the clinic. Its simplicity, standardization and potential for patient-specific approaches make it an attractive option for developing cell-based therapies. The use of small molecules also facilitates regulatory approval processes by providing a more controlled and defined approach to reprogramming. These factors contribute to the potential clinical translation of chemical transdifferentiation, bringing regenerative medicine closer to becoming a reality for patients in need.

Chemical transdifferentiation represents a game-changing approach in the field of regenerative medicine. Although challenges lie ahead and further research is urgently needed, the advantages of chemical transdifferentiation has the power to revolutionize the way we approach tissue regeneration, disease modeling and personalized therapies.

### 3.2. Unlocking Potential: Transdifferentiation of Various Cell Types

The use of small molecules to induce somatic cell transdifferentiation offers immense possibilities for regenerative therapies and holds the potential to revolutionize the field of medicine. Here are some examples of small molecules that have been used to promote cell transdifferentiation (Table 2). The effectiveness of small molecules varies according to specific experimental conditions, cell types and other factors used in conjunction with them.

(1) Fibroblasts to Neurons: One of the most striking achievements in chemical transdifferentiation is the conversion of fibroblasts, which are common connective tissue cells, into functional neurons. Previous study showed that small molecules, such as valproic acid (VPA), forskolin and CHIR99021, could modulate the signaling pathways involved in neuronal development. Via chemical screening, Li et al. identified that the combination of forskolin, ISX9, CHIR99021 and I-BET151 could convert mouse fibroblasts into neuronal cells, with a yield of up to >90% being TUJ1-positive after 16 days of induction. After a further mature cultivation, the chemically induced neurons possessed neuron-specific expression patterns, generated action potentials and formed functional synapses. Among the molecules, I-BET151, a BET family bromodomain inhibitor, disrupted the fibroblast-specific program and the neurogenesis inducer ISX9 was necessary to activate neuron-specific genes [41]. Through the activation of specific genes and the rewiring of cellular signaling networks, fibroblasts were transformed into functional neurons, providing new avenues for the treatment of neurodegenerative disorders and nerve injuries [41,54,55].

(2) Fibroblasts to Cardiomyocytes: Small molecules have also been demonstrated to reprogram fibroblasts into beating cardiomyocytes. By using a series of small molecules, such as PD0325901, CHIR99021 and A83-01, researchers successfully induced the expression of cardiac-specific genes and facilitated the morphological and functional transformation of fibroblasts into cardiomyocytes [19,42]. During this process, small molecule treatment resulted in a more open chromatin conformation at key heart developmental genes, enabling their promoters and enhancers to bind effectors of major cardiogenic signals. When the chemically induced cells were transplanted into the hearts of infarcted mice, they exhibited well-organized sarcomeres and partially remuscularized the infarcted areas [19]. This breakthrough holds tremendous potential for cardiac tissue regeneration and the development of personalized therapies for heart diseases. 

(3) Fibroblasts to Hepatocytes: Hepatocyte transplantation is a promising alternative to whole-organ transplantation to support many forms of liver failure; thus, transdifferentiation of fibroblasts into hepatocytes is of great therapeutic interest. However, researchers have not yet derived fully chemical hepatocytes from fibroblasts, but their studies provide us valuable information that the small molecules CHIR99021, A83-01, VPA, HGF et al. facilitate factor-mediated hepatic transdifferentiation [44,45]. In 2017, Guo et al. converted mouse fibroblasts into hepatocyte-like cells by chemical cocktails in combination with a single transcription factor. The derived cells had typical epithelial morphology, expressed multiple hepatocyte-specific genes and could reconstitute the damaged hepatic tissues of fumarylacetoacetate hydrolase-deficient (Fah^−/−^) mice [44]. Although some investigations also showed that functional hepatocytes could be derived from fibroblasts by ectopic expression of transcription factors, it is still worthy of efforts to obtain transcription factor-free hepatocytes which might be more amendable in clinical applications.

(4) Fibroblasts to Pancreatic Beta Cells: The conversion of fibroblasts into insulin-producing pancreatic beta cells holds immense promise for diabetes treatment and research. Small molecules, such as Activin A, CHIR99021 and epigenetic modulators NaB and RG108, have been utilized to trigger the transdifferentiation process by modulating the development and gene expression of pancreatic beta cells [49,51]. This approach has shown success in generating functional beta-like cells that can secrete insulin in response to glucose levels. In addition, the small molecule harmine has been found to promote the conversion of human pluripotent stem cells and other cell types into beta-like cells by activating the Pdx1 pathway, which plays a critical role in pancreatic development and beta cell function [52,53]. 

(5) Fibroblasts to Endothelial Cells: Using small molecules, Sayed et al. derived functional endothelial cells from human fibroblasts for the first time in 2015 [46]. Cognizant of the role of innate immunity in nuclear reprogramming, they used PolyI:C, a Toll-like receptor 3 agonist, to activate innate immunity, followed with microenvironment-based induction to endothelial lineage [46,48]. The derived endothelial cells expressed endothelial-specific markers, took up low-density lipoprotein, secreted angiogenic cytokines under hypoxic conditions and formed microvessels in vitro and in vivo. Our recent study showed that although p53 expression level did not change during cardiac fibroblast–endothelial cell transition, p53 activation facilitated this transition, which mainly functioned in the later stage of endothelialization. 

In addition to fibroblast-based transdifferentiation, small molecules are also used to induce cellular plasticity between different lineages [51,56,57,58]. For instance, researchers have successfully converted epithelial cells, which are located on the surface of the skin and lining of internal organs, into mesenchymal cells [56]. This epithelial–mesenchymal transition (EMT) can be triggered by a combination of small molecule inhibitors targeting TGF-β and histone deacetylase inhibitors. Furthermore, a combination of small molecules including VPA, CHIR99021 and RepSox has been used to convert astrocytes into functional neurons [57]. These small molecules modulate key signaling pathways involved in neuronal development and facilitate the conversion. In response to injury, a reparative process is triggered to restore the damaged tissue [59]. This process is sometimes accompanied by cell transdifferentiation, which is a spontaneous process stimulated by pathological factors, such as myocardial fibrosis and retinal fibrosis. These lesions often lead to organ dysfunction, so timely intervention is necessary. Recently, Sloan et al. investigated the effects of N-oleoyl dopamine and identified its function on TGF-β-induced myofibroblast transdifferentiation of retinal pigment epithelial cells, indicating its therapeutic value for treating fibrotic pathologies [60]. The versatility and potential of chemical transdifferentiation are rapidly expanding as scientists continue to explore new combinations of small molecules and refine the mechanisms driving cell fate conversion. By unlocking the potential to convert one cell type into another, this technique offers a new frontier for personalized therapies, disease modeling and tissue engineering. 

### 3.3. Challenges: The Bottleneck of Chemical Transdifferentiation 

Although chemical transdifferentiation holds immense potential, there are still challenges and limitations hindering its clinical application. Here, we will explore the major hurdles that researchers face and discuss the current efforts to overcome these obstacles. 

One of the primary challenges in chemical transdifferentiation is achieving high conversion efficiency and reproducibility [61]. The process of reprogramming cells using small molecules is inherently complex and context-dependent. Identifying an optimal combination of small molecules, their concentrations and treatment durations for specific cell types requires extensive experiment. Additionally, the efficiency of chemical transdifferentiation varies across different cell types, different cell sources, et al. Although a combination of small molecules successfully converted certain cells, it might not work on the others [62,63]. As Trokovic et al. suggested, reprogramming efficiency correlated negatively and declined rapidly with increasing donor age [62]. Researchers continue to explore new strategies and techniques to overcome these barriers, including the identification of novel regulating factors and the refinement of existing protocols.

Understanding the molecular mechanisms driving cell transdifferentiation is necessary. Although researchers have successfully induced cell fate changes using small molecules, the underling mechanisms by which these molecules initiate and facilitate the conversion are not fully elucidated [64,65]. Further research is needed to unravel the intricate molecular pathways and regulatory networks involved in the transdifferentiation, which will contribute to the development of more efficient small molecule cocktails, enhancing the efficacy of the process [66].

Ensuring the long-term stability and functionality of transdifferentiated cells remains a critical concern [67]. Factors such as epigenetic memory, incomplete conversion and potential dedifferentiation back to the original cell type pose obstacles to the stability and functionality of transdifferentiated cells [67,68]. In order to eliminate the obstacles, researchers focus on optimizing small molecule cocktails and manipulating cellular environments. In fact, thoroughly evaluating the chemically induced cells compared to their natural counterparts is also necessary because ensuring that the transdifferentiated cells closely resemble their naturally occurring counterparts in terms of phenotype, function and gene expression profiles is essential for their safe and effective use [69]. However, current methods of chemical transdifferentiation may not always generate fully mature and functional cells, leading to the need for further optimization and characterization [62,70].

The scalability and clinical translation of chemical transdifferentiation pose significant challenges. While small molecules are generally easier to synthesize and deliver than other reprogramming methods, the process of large-scale production and quality control may still present obstacles. Additionally, translating chemical transdifferentiation from the laboratory to clinical applications requires rigorous safety and efficacy testing, regulatory approvals and considerations for manufacturing and delivery methods.

Chemical transdifferentiation holds immense promise in the field of biomedicine, while several challenges hinder its clinical application. Overcoming the limitations requires interdisciplinary collaborations, innovative approaches and continued research efforts. 

## 4. Future Perspectives of Chemical Transdifferentiation

**Figure 2:**
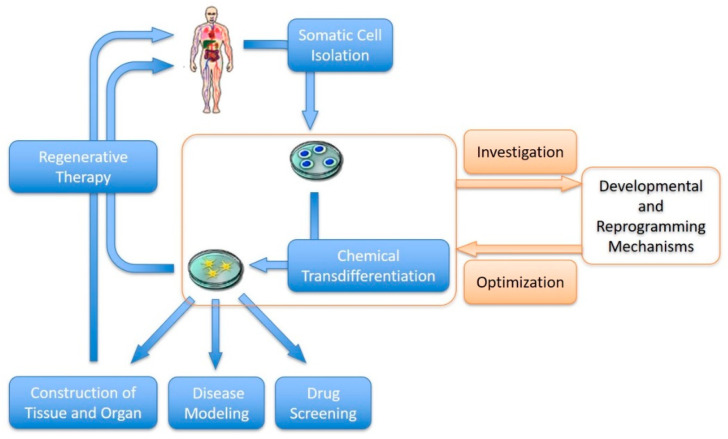
Perspectives of chemical transdifferentiation in biomedicines.

In this review, we focus on the development of the chemical transdifferentiation of somatic cells and discuss its advantages over other transdifferentiation methods and challenges hindering its clinical application. The future of chemical transdifferentiation is bright, with the potential to significantly impact various fields, including regenerative medicine, disease modeling, drug discovery and tissue engineering (Figure 2). Chemical transdifferentiation enables direct conversion of patient-specific somatic cells into desired cell types for replacement therapy, which will offer new treatment options for a wide range of diseases, including neurodegenerative disorders, cardiovascular conditions and organ regeneration [19,41,71]. Additionally, chemical transdifferentiation can be used to generate disease-specific cell types, providing valuable tools for disease modeling and drug screening. These cells can be used to study disease mechanisms, test potential therapeutic interventions and identify new drug targets [69]. Chemical transdifferentiation also holds potential in tissue engineering and organogenesis [72]. By converting somatic cells into specific cell types, it is possible to generate functional tissues and organs in the laboratory, addressing the shortage of donor organs for transplantation. In addition, the process and mechanism of cell transdifferentiation have significant research value, which will expand our understanding of developmental mechanisms and pathological processes [73].

## 5. Conclusions

In this review, we summarized the current progress and future perspective of the chemical transdifferentiation of somatic cells and discussed its potential impact on biomedicine and the limitations hindering its clinical application. With ongoing research and technological advancements, the future holds tremendous promise for harnessing the power of small molecules to shape the cellular landscape and revolutionize the field of biomedicine. 

## Figures and Tables

**Table 1:** Comparisons of some methods for cell transdifferentiation.

Method	Advantages	Shortcomings
Transcription Factor Overexpression	- Direct and specific reprogramming of cells - Well-established techniques - High efficiency in some cases	- Requires genetic manipulation - Limited to cell types with known transcription factor combinations - Potential risk of tumorigenicity
Small Molecules	- Non-genetic approach - Can be easily delivered to target cells - Versatile and modifiable	- Less efficient compared to transcription factor-based approaches - Requires optimization and identification of specific molecules
MicroRNA-Based Reprogramming	- Non-genetic approach - Fine-tuned regulation of gene expression	- Complex interaction networks between microRNAs and target genes - Limited efficiency
EV-Based Conversion	- Utilizes environmental cues and signaling factors - Mimics developmental processes	- Limited efficiency and reproducibility - May require complex and expensive culture conditions
Three-Dimensional Culture Systems	- Provides a more physiological context - Better recapitulation of tissue architecture and function	- Complexity in establishing and maintaining 3D cultures - Variability in differentiation outcomes

**Table 2:** Representative small molecules that have been suggested to promote somatic cell transdifferentiation.

Transdifferentiation	Small Molecules	Mechanism	References
Fibroblasts to Neurons	Valproic acid (VPA)	Histone deacetylase (HDAC) inhibitor	[40]
	Forskolin	Activator of the cyclic adenosine monophosphate (cAMP) pathway	[41] [40]
	ISX9	Stimulates neurogenesis	[41]
	CHIR99021	Inhibitor of glycogen synthase kinase 3 beta (GSK3β)	[41] [40]
	Repsox	Inhibitor of transforming growth factor-beta (TGF-β) receptor	[40]
Fibroblasts to Cardiomyocytes	CHIR99021	Inhibitor of GSK3β	[19] [42]
	A83-01	Inhibitor of TGF-β type I receptor	[19]
	LIF	Leukemia inhibitory factor	[42]
	PD0325901	Inhibitor of MEK1/2	[42]
	Y-27632	ROCK inhibitor	[19]
	AS8351	Histone demethylase (HDM) inhibitor	[19]
	SU16F	PDGFRβ inhibitor	[19]
Fibroblasts to Hepatocytes	A83-01	Inhibitor of TGF-β type I receptor	[43]
	VPA	HDAC inhibitor	[44] [45]
	CHIR99021	Inhibitor of GSK3β	[44] [45] [43]
	HGF	Hepatocyte growth factor	[43]
	EGF	Epidermal growth factor	[43]
	TTNPB	Retinoic acid (RA) receptor agonist	[44] [45]
	Dznep	Histone methyltransferase EZH2 inhibitor	[44] [45]
Fibroblasts to Endothelial Cells	VEGF	Vascular endothelial growth factor	[46] [47]
	SB431542	Inhibitor of TGF-β receptor	[46] [47]
	bFGF	Basic fibroblast growth factor	[46] [47]
	BMP4	Bone morphogenetic protein 4	[46] [47]
	PolyI:C	Toll-like receptor 3 (TLR3) agonist	[46] [47]
	RITA	Inhibitor of the p53-MDM2 interaction	[48]
	8-Br-cAMP	Activator of cyclic AMP-dependent protein kinase	[47]
Fibroblasts to Pancreatic Beta Cells	Activin A	Member of TGF- β superfamily	[49]
	Forskolin	Activator of the cAMP pathway	[50] [49]
	GDC-0449	Antagonist of sonic hedgehog	[49]
	Nicotinamide	Vitamin B3 or niacin	[50] [49]
	Sodium butyrate (NaB)	Inhibitor of histone deacetylase	[51] [49]
	RG108	Inhibitor of DNA methylase	[51] [49]
	Compound-E	Inhibitor of Notch signaling	[49]
	Harmine	Inhibitor of DYRK1A	[52] [53]
	Dexamethasone	Agonist of glucocorticoid receptor	[50] [49]

## Data Availability

No new data were created or analyzed in this study. Data sharing is not applicable to this article.
